# Handling of diet-related risks in psychosomatic rehabilitation clinics

**DOI:** 10.1192/j.eurpsy.2025.1205

**Published:** 2025-08-26

**Authors:** R. Hiltensperger, J. Neher, L. Böhm, A. S. Mueller-Stierlin

**Affiliations:** Institute for Epidemiology and Medical Biometry, University of Ulm, Ulm, Germany

## Abstract

**Introduction:**

People with mental health conditions frequently encounter diet-related challenges that contribute to the onset of physical comorbidities, further diminishing their quality of life and life expectancy. To date, diet-related problems often are not adequately addressed in mental health care for reasons like overshadowing.

**Objectives:**

Psychosomatic rehabilitation clinics are a potential setting for tackling these problems as the admission takes place beyond acute crisis and as multi-professional teams, including dietitians, are available. However, little is known so far about the extent to which this potential is being exploited.

**Methods:**

In summer 2024, we conducted 22 qualitative interviews with physicians, dietitians and other staff members at six psychosomatic rehabilitation clinics in Germany to explore the current state of action in handling diet-related risks in psychosomatic rehabilitation clinics.

**Results:**

Interviewees elaborated on their eexperiences with the prescription and utilization of diet-related interventions in psychosomatic rehabilitation and their subjective assessments of the potential added value of screening tools in care planning. Preliminary analyses indicate that diet-related problems have not yet been systematically captured in the rehabilitation process. Due to a lack of time, a comprehensive nutrition-related assessment is hardly possible and therefore the diet-related activities tend to be one-size-fits-all services.

**Conclusions:**

The findings of the qualitative study endorse the implementation of a multi-faceted screening tool, such as the NutriMental screener. By this way, diet-related problems of service users might be recognized at the beginning of inpatient rehabilitation and being addressed appropriately during the inpatient stay by selecting suitable rehabilitation measures, especially dietary interventions.

**Disclosure of Interest:**

None Declared

